# Perinatal vulnerability: its impact on oral and craniofacial hard tissue development

**DOI:** 10.3389/fphys.2026.1773684

**Published:** 2026-05-05

**Authors:** Takehito Ono

**Affiliations:** Laboratory of Drug Discovery and Pharmacology, Faculty of Veterinary Medicine, Okayama University of Science, Ehime, Japan

**Keywords:** development, low birth weight (LBW), oral and craniofacial deformity, preterm birth, small for gestational age (SGA)

## Abstract

Advances in perinatal and neonatal medicine have dramatically improved the survival rates of infants with perinatal vulnerabilities of preterm birth (PTB), low birth weight (LBW) or small for gestational age (SGA), by rescuing these infants from immediate postnatal complications. Despite improved survival from such problems, it has in turn highlighted long-term complications, including psychiatric, neurological, respiratory, cardiovascular and metabolic diseases. In the oral and craniofacial regions, perinatal vulnerability is associated with an increased risk of developmental complications, including bone deformities, malocclusion and tooth malformations. These anomalies are often tangible and can be recognized earlier than extracranial sequelae; thus, they may serve as accessible indicators for predicting extracranial sequelae that remain latent. Oral and craniofacial sequelae are considered consequences of changes in nutritional status and oxygen saturation before and after birth, which are related to epigenetic changes. Additionally, the development of oral and craniofacial tissues is controlled by mechanical forces. The underlying mechanisms have not been fully elucidated, and future studies elucidating these mechanisms will enhance diagnostic and therapeutic strategies, as well as prognostic accuracy, ultimately improving long-term outcomes for infants born with perinatal vulnerabilities.

## 1 Introduction

Reproduction is a biological process that unites male and female gametes, combining their genetic information to create individuals of the next generation with a variety of genetic profiles. This genetic diversity enhances the survival of the species in response to environmental changes. Indeed, this mode of reproduction, namely sexual reproduction, has enabled the persistence of the human species today. However, for a single-celled zygote to develop into a fetus, gestation and labor processes must be supported by complex endocrine and immune regulation.

Disruptions in the regulation of gestation/labor processes may shorten or prolong gestation, whereas impaired nutrient and oxygen supply can lead to fetal growth restriction (FGR), which can lead to low birth weight (LBW). Infants with such perinatal vulnerabilities exhibit complexities during labor. With the development of the neonatal intensive care unit (NICU) infrastructure and the newborn emergency transportation system (NETS), there has been a significant improvement in the survival rate of infants with complexities associated with perinatal vulnerabilities (Hajdu et al., 2024; Monzon et al., 2023; Owen et al., 2017).

Although the enhancement of neonatal medicine has significantly improved the survival of infants with perinatal vulnerabilities, accumulated evidence has revealed that there are not only short-term but also long-term complications. These include sequelae of neurodevelopmental, pulmonary, cardiovascular, immune and endocrine disorders, as well as those of the bones and teeth, which can coincide through the interaction of underlying signaling pathways.

These sequelae extend beyond the individual, profoundly affecting families and communities, posing not only medical challenges but also significant socioeconomic burdens. This review summarizes the oral and craniofacial developmental complications in these individuals, focusing on their mechanisms of pathogenesis, and potential approaches to developmental care based on these underlying mechanisms, with the aim of advancing perinatal medicine.

## 2 Normal process of gestation and labor

Gestation and labor are pivotal events in the process of giving birth to the next generation, by growing a single cell into a fetus in the uterus, through the germinal, embryonic and fetal stages.

### 2.1 Classification of gestation period

The gestation process begins when the sperm fertilizes the ovum in the Fallopian tubes. The fertilized ovum, zygote, undergoes cell division to form a blastocyst in the uterus. On 7–8 days after fertilization, it invades into the endometrium (*i.e.* implantation) to establish gestation. The maternal-germinal interaction forms the placenta, enabling the nutrition and oxygen supply to the offspring (Erlebacher, 2013; Oliver and Basit, 2023).

During the embryonic stage (3–8 post-conceptional weeks; pcw), the three germ layers (ectoderm, mesoderm and endoderm) become distinguishable, leading to the formation of various tissues and organs, and establishing the basic structure of the body (Haniffa et al., 2021). Given that this period represents a critical window for organogenesis, infectious or chemical stimuli exert serious effects on organ development (Haniffa et al., 2021; Srivastava et al., 2023).

After the embryonic stage, the embryo, which begins to be called the fetus, undergoes a further growth stage (the fetal period: 9 pcw and later), in which tissues and organs mature, and the size of the fetus increases rapidly (Haniffa et al., 2021).

### 2.2 Clinical classification of gestation period

Clinically, the embryonic and fetal stages are split into 3 trimesters; first: 13 weeks of gestation and earlier, second: 14–26 weeks of gestation and third: 27 weeks of gestation and later; counted from the last menstrual period (LMP), approximately 2 weeks before pcw.

### 2.3 Process of labor

**Figure 1:**
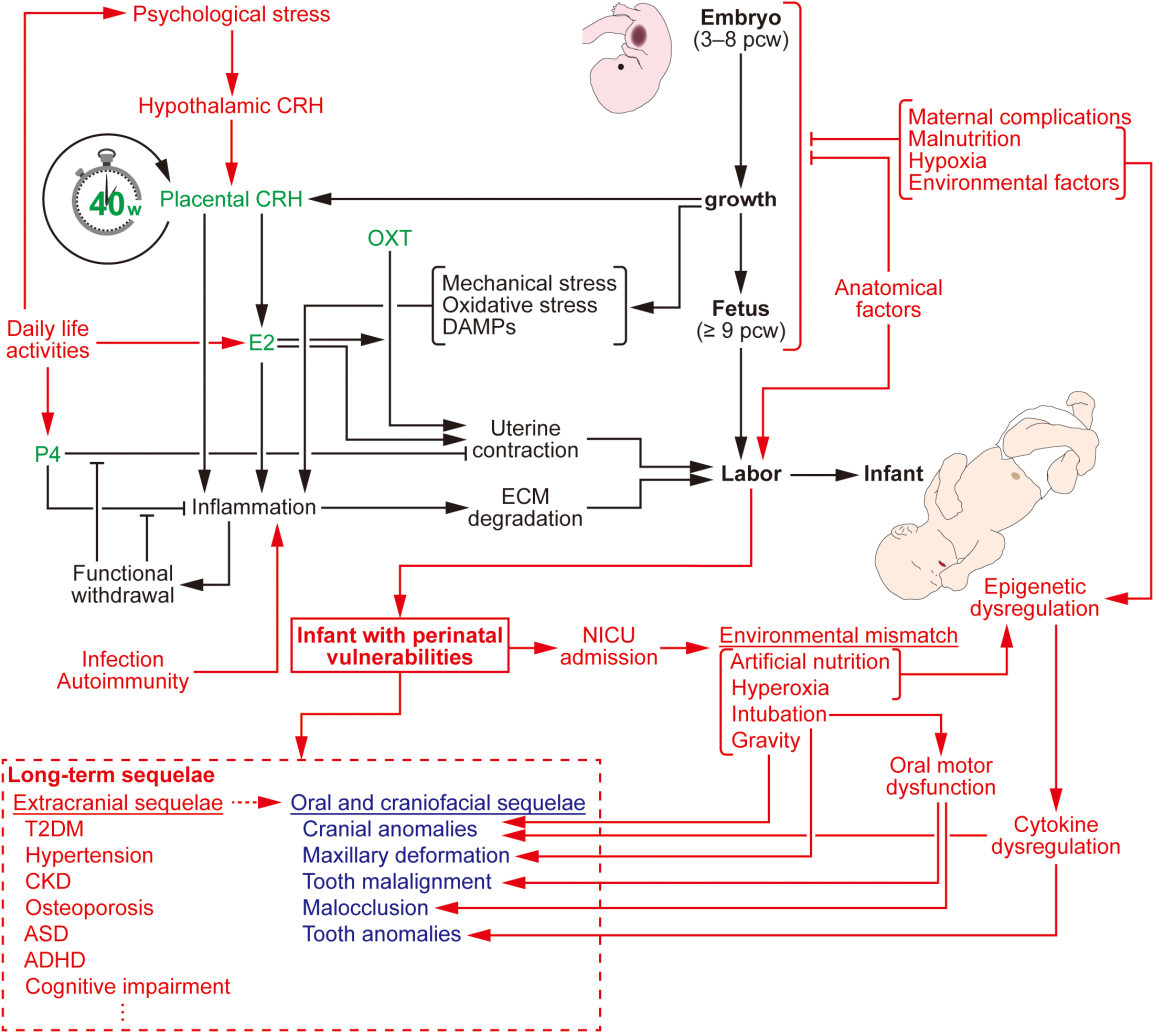
Gestation/labor process and perinatal vulnerabilities. Human gestation is strictly regulated to approximately 40 weeks by hormonal factors. The concentration of these hormones rises as the fetus develops *in utero*. Signals from the developing fetus, as well as these hormones, elicit sterile inflammation in the uterus, driving the labor process. However, disruption of this process by internal or external factors can result in perinatal vulnerabilities and subsequent long-term sequelae. Notably, even treatments in the NICU can disturb normal development of the infant and contribute to these sequelae. These long-term outcomes manifest in the oral and craniofacial region, as well as in extracranial regions. Black texts and lines depict the physiological process of gestation/labor. Green texts denote essential hormones regulating this process. Red text and lines indicate abnormal stimuli that can induce perinatal vulnerabilities. Blue texts highlight long-term sequelae in the oral and craniofacial region. ADHD, attention deficit hyperactivity disorder; ASD, autism spectrum disorder; CKD, chronic kidney disease; CRH, corticotropin-releasing hormone; DAMPs, damage-associated molecular patterns; E2, estradiol; ECM, extracellular matrix; NICU, neonatal intensive care unit; OXT, oxytocin; pcw, post-conception weeks; P4, progesterone; T2DM, type 2 diabetes mellitus.

Normal labor is the process of delivering a viable fetus *ex utero*. In humans, infants are delivered at 39 and 40 weeks of gestation. To prevent this process from occurring before term, the myometrium exerts minimal contraction force, and the uterine outlet, cervix, forms a rigid barrier of dense extracellular matrix (ECM). These mechanisms of containing the fetus are regulated by various factors, as mentioned below (**Figure 1**).

#### 2.3.1 Hormonal regulation

Progesterone (P4) suppresses spontaneous uterine contractions during pregnancy. In contrast, in the late gestational phase, intracellular P4 is degraded and its signal is inhibited by PR-A. This “functional withdrawal” of P4 is induced by local inflammation and in turn augments the inflammation. This further induces the expression of Cx43, a gap junction protein that synchronizes myometrial contractions (Hamburg-Shields and Mesiano, 2024; Tan et al., 2012), together prompting fetal expulsion.

Corticotropin-releasing hormone (CRH) is mainly released from the hypothalamus to drive the hypothalamic-pituitary-adrenal (HPA) axis, a stress response signal cascade (Claes, 2004). In the process of gestation and labor, it is highly expressed by positive feedback loop in the placenta during the last trimester, thus being used for predicting the timing of birth as “the placental clock” (McLean et al., 1995). Placental CRH induces the synthesis of estrogens in the placenta using DHEA-S produced in the fetus and mild inflammation in the uterus (Vrachnis et al., 2012).

One of the estrogens, estradiol (E2) has crucial roles in labor, as well as preparing and maintaining pregnancy. Upon labor, it induces matrix metalloproteinase (MMP)-2 and MMP-9, which degrade the ECM in the cervix and amnion, *via* inflammation (Hamburg-Shields and Mesiano, 2024; Stygar et al., 2002). Furthermore, E2 increases the expression of Cx43, the oxytocin receptor (OXTR) and prostaglandin receptors, thereby enhancing uterine contraction (Hamburg-Shields and Mesiano, 2024). The expression of Oxytocin (OXT) and OXTR increases during pregnancy and peaks at labor to induce uterine contractions to deliver the fetus (Uvnas-Moberg, 2024).

#### 2.3.2 Inflammatory regulation

The immune system can provoke inflammation without infection, known as sterile inflammation (Gong et al., 2020). This occurs during gestation and labor, which is increasingly recognized as critical in these contexts. As gestation proceeds, mechanical and oxidative stresses increase, and more damage-associated molecular patterns (DAMPs), including cell-free fetal DNA (cffDNA) are released in circulation. DAMPs are recognized by innate immune receptors such as Toll-like receptors (TLRs) and receptor for advanced glycation end-products (RAGE) to induce pro-inflammatory cytokines (Goldfarb et al., 2018; Negishi et al., 2021). These cytokines further induce prostaglandins (PGs), PGE_2_ and PGF_2α_ and their receptors, which induce uterine contractility, collagen degradation and proteoglycan synthesis (Sykes et al., 2014). Inflammation is also known to drive the expression of OXTR, PR-A and Cx43 (Leimert et al., 2021). Thus, hormonal and inflammatory factors interact synergistically to regulate the timing of birth.

## 3 Evaluation of perinatal vulnerability

Developed through these processes, infants are normally born at approximately 40 weeks of gestation, with a body weight of approximately 2,500-4,000 g. Infants outside this range may face short and long-term complications. Perinatal vulnerability is assessed mainly by gestational period and/or birth weight, as these parameters are closely related to the complications of perinatal vulnerability. The following parameters are most commonly used, either alone or in combination.

### 3.1 Classification of perinatal vulnerability by gestation period

Human development progresses through precisely timed organ formation and maturation, as described above. Therefore, assessing gestational age is critical for predicting organ-specific abnormalities that underpin perinatal vulnerabilities. The optimal human gestational period is defined as 39 0/7 weeks - 40 6/7 weeks, which is referred to as “full term,” as the frequency of adverse neonatal outcomes is the lowest. The periods outside the range of the full term are listed in **Table 1**. Approximately 10% of children worldwide, nearly 15 million children in total, are born before the 37th week of gestation (Deindl and Diemert, 2020; Ohuma et al., 2023), with immature physiology.

**Table 1:** Classification of perinatal vulnerability.

Classification of deliveries by gestational age	
Preterm	36 6/7 weeks and before
Extremely preterm (EPT)	27 6/7 weeks and before
Very preterm (VPT)	28 0/7 weeks through 31 6/7 weeks
Moderate preterm	32 0/7 weeks through 33 6/7 weeks
Late preterm	34 0/7 weeks through 36 6/7 weeks
Early term	37 0/7 weeks through 38 6/7 weeks
Full term	39 0/7 weeks through 40 6/7 weeks
Late term	41 0/7 weeks through 41 6/7 weeks
Post term	42 0/7 weeks and over
Classification of deliveries by body weight	
Low birth weight (LBW)	lower than 2,500 g
Extremely low birth weight (ELBW)	lower than 1,000 g
Very low birth weight (VLBW)	higher than/equal to 1,000 g and lower than 1,500 g
Normal birth weight	2,500 g through 4,000 g
Macrosomia	higher than 4,000 g
Classification by the combination of gestational age and body weight (originate from Lubchenco curve)	
Small for gestational age (SGA)	below 10th percentile
Average for gestational age (AGA)	10th through 90th percentile
Large for gestational age (LGA)	over 90th percentile
Fenton chart	
Gestational age vs. birth weight	
Small for gestational age (SGA)	below 10th percentile
Average for gestational age (AGA)	10th through 90th percentile
Large for gestational age (LGA)	over 90th percentile
Gestational age vs. birth head circumference	
Small for gestational age (SGA)	below 10th percentile
Average for gestational age (AGA)	10th through 90th percentile
Large for gestational age (LGA)	over 90th percentile
Gestational age vs. birth length	
Small for gestational age (SGA)	below 10th percentile
Average for gestational age (AGA)	10th through 90th percentile
Large for gestational age (LGA)	over 90th percentile

This table categorizes perinatal vulnerabilities by gestational age, birth weight and size for gestational age using the Lubchenco curve and Fenton chart.

### 3.2 Classification of perinatal vulnerability by birth weight

Although it correlates with gestational age to some extent, body weight at birth is another independent indicator of perinatal vulnerability. Infants are usually born with a body weight of 2,500–4,000 g. Based on this, birth weight is classified as shown in **Table 1**. Approximately 20 million or 15% of liveborn newborns are reported to have LBW, with a decreasing tendency from 2000 due to efforts to improve the nutrition of the fetus (Okwaraji et al., 2024).

PTB is one of the primary drivers of LB; however, term infants can also experience this type of perinatal vulnerability in cases of fetal growth restriction (FGR), also known as intrauterine growth restriction (IUGR). FGR is assessed using the estimated fetal weight (EFW) derived from ultrasound measurements; an EFW below the 10th percentile leads to a diagnosis of FGR (Society for Maternal-Fetal Medicine (SMFM) et al., 2020).

### 3.3 Classification of perinatal vulnerability by body length at birth

Birth length is a readily measurable parameter at birth; however, it is rarely used alone to assess perinatal vulnerability and is typically combined with other parameters (*see below*).

### 3.4 Classification of perinatal vulnerability by head circumference at birth

Head circumference (HC) is closely related to the genetic background of infants, and each country has its own calibrated criterion (Bergerat et al., 2021). Considering that the cranium encases the brain, it is intuitive to assume that this parameter is associated with neurodevelopmental outcomes.

### 3.5 Classification of perinatal vulnerability using multiple parameters

No single parameter can fully assess the risk of complications in infants with perinatal vulnerability. Currently, the Fenton chart (developed in 2003 and revised in 2013) is widely used for evaluating preterm infants by plotting birth weight, length and head circumference (Fenton and Kim, 2013) (**Table 1**). This curve facilitates visualization of infant growth, allowing clinicians to promptly identify growth abnormalities and appropriately guide nutritional interventions. Such visualization also aids in educating parents about their infant’s growth patterns and the need for medical intervention.

## 4 Risks, causes and outcomes of perinatal vulnerabilities

Epidemiologically, the incidences of PTB and LBW are particularly high in Southern Asia and sub-Saharan Africa, which are primarily attributed to socioeconomic disparities, rather than genetic factors (Gette et al., 2025; Zhou et al., 2025). Studies have revealed that perinatal vulnerability is a multifactorial condition, rather than a monocausal disease (**Figure 1**).

### 4.1 Risk factors and causes for PTB

As described above, gestation and labor are regulated by an intricate interplay of hormonal and inflammatory pathways, and the dysregulation of even a single element contributes to PTB.

#### 4.1.1 Anatomical factors

Fetal retention is influenced by anatomical variations and abnormalities in pregnant women, especially in the reproductive organs. Maternal body type, ethnicity, previous PTB and previous miscarriage contribute to the risk of PTB because they are associated with reproductive organ morphology (Goldenberg et al., 2008). In addition, gestational period can be compromised by short cervix, cervical insufficiency, premature cervical dilation, Müllerian anomalies and surgeries of the uterus (including the cervix) (Yan et al., 2023).

#### 4.1.2 Endocrine and neurovascular dysregulations

Alteration of hormonal signals can influence the birth period, even with non-pathological fluctuations. Solar exposure can increase the serum concentrations of P4 and E2, suggesting that daily life activities can influence the gestational period (Parikh et al., 2021). Pregnant women may experience psychological stress, which can drive the HPA axis and the placental positive feedback loop of CRH to prompt labor (Najafzadeh, 2016). Psychological stress can also activate the sympathetic-adrenal-medullary (SAM) axis to secrete catecholamines. Noradrenaline strongly induces the contractions of blood vessels in the placenta and subsequent PTB by disturbing circulation in the placenta (Najafzadeh, 2016). Environmental heat stress is associated with maternal hypertension, which increases the risk of PTB (Wang et al., 2024b). The activities of the neuronal and endocrine systems are affected by chemicals and medications; in particular, the use of tobacco, alcohol and psychoactive substances increases the risk of PTB (Salam and Mitchell, 2022).

#### 4.1.3 Infection and inflammation

During gestation, inflammation can be elicited in contexts other than at the beginning of labor. One of the most prominent inflammatory conditions that causes PTB is chorioamnionitis (CAM), which is elicited by many types of bacteria, as well as by damage to the placental tissues (*i.e.* sterile inflammation) (Negishi et al., 2022).

Inflammation of the tissues outside the placenta can also induce PTB. Autoimmune diseases, such as systemic lupus erythematosus (SLE), antiphospholipid antibody syndrome (APS) and type I diabetes (T1DM), are known to increase the risk of PTB by inducing a certain level of systemic inflammation, by impairing placental blood flow and inducing CRH (Kolstad et al., 2020; Murphy et al., 2021; Negro, 2011). Glucocorticoid therapy can lead to PTB (Shimada et al., 2022). Periodontitis, the most common infectious disease in humans, can affect the placenta, although it is distant from the primary lesion, raising the risk of PTB (Vergnes and Sixou, 2007; Yan et al., 2023). Inflammation induced by type II DM (T2DM) and senescence (*i.e.* inflammaging) can influence the gestational period as well (Clement et al., 2025; Zavatta et al., 2023).

### 4.2 Risk factors and causes for FGR

FGR is attributed to disorders of the maternal, placental and fetal components; malnutrition, hypoxia and/or metabolic dysregulation of the fetuses are considered shared processes.

#### 4.2.1 FGR of maternal origin

Respiratory diseases and inadequate nutrition of the pregnant woman impair the supply of oxygen and nutrition to the fetus, respectively. Circulatory diseases can compromise both. Infectious and several non-communicable (*e.g.* autoimmune and metabolic) diseases elicit inflammation in the maternal compartment, which results in endothelial dysfunction, leading to poor blood supply (Sharma et al., 2016).

#### 4.2.2 FGR of placental origin

Vascular insufficiency in the placenta compromises the supply of both oxygen and nutrition to the fetus. The placentae of FGR fetuses are smaller than non-FGR placentae, with less vasculature inside, thus having a limited exchange capacity for nutrition and oxygen (Sun et al., 2020). Genetic diseases and epigenetic dysregulation are thought to result in placental insufficiency (Shi et al., 2022; Song et al., 2024).

#### 4.2.3 FGR of fetal origin

Certain genetic or teratogenic inputs can lead to congenital malformations that result in FGR. These include chromosomal numerical abnormalities, copy number variants, genetic diseases and epigenetic changes (Shi et al., 2022). Even in the absence of such disorders, in the case of multiple gestations, there will be competition for nutrient or oxygen resources, increasing the risk of FGR and subsequent LBW (Sharma et al., 2016).

### 4.3 Systemic outcomes of perinatal vulnerabilities

If an infant is delivered with perinatal vulnerabilities due to genetic disorders, its postnatal development is compromised, as dictated by the underlying mutations. On the other hand, perinatal vulnerability can affect development without evident genetic causes.

#### 4.3.1 Metabolic maladaptation by perinatal vulnerabilities

Infants born with LBW or delivered preterm experience different hormonal (withdrawal from maternal circulation), nutritional and oxygen conditions (including hospitalization in the NICU) from those with normal birth weight and delivered full term. To survive under such conditions, adaptation of the metabolic program occurs in fetuses and infants. This metabolic reprogramming can be irreversible and can continue to function lifelong (the Developmental Origins of Health and Disease [DOHaD] hypothesis); the changed metabolic program may not match the circumstances in adulthood. These mismatches can lead to various diseases in adulthood (Gluckman and Hanson, 2004a, b).

#### 4.3.2 Diseases associated with perinatal vulnerabilities

Studies have confirmed that LBW increases the risk of non-communicable diseases (NCDs), such as T2DM, hypertension, dyslipidemia, proteinuria, chronic kidney disease (CKD) and osteoporosis (Bianchi and Restrepo, 2021; Del Real et al., 2018; Itoh and Kanayama, 2015; Yoshida-Montezuma et al., 2023). Under nutrient-restricted conditions, nutrition is preferentially allocated to the central nervous system (CNS), leading to a reduced nutrient supply to peripheral organs and subsequent diseases described above. However, this protective system of the brain (brain sparing) does not seem to completely protect the brain against poor nutrition, as individuals with perinatal vulnerabilities would suffer from neuropsychiatric disorders, such as autism spectrum disorder (ASD), attention-deficit hyperactivity disorder (ADHD) and poor cognitive development, which are closely related to their HC (Aagaard et al., 2018; Benitez-Marin et al., 2021; Cirnigliaro et al., 2024).

#### 4.3.3 Epigenetic alteration in perinatal vulnerabilities

The adaptive reprogramming of fetuses and infants is thought to be in part under epigenetic regulation. The intake of macronutrients, including carbohydrates, lipids and proteins, can alter the methylation status of certain genetic loci in the offspring, thereby influencing their metabolic profiles and potentially increasing their susceptibility to diseases (Bogdarina et al., 2007; Hor et al., 2025). It has been reported that not only these nutrients, but also environmental factors (*e.g.* smoke) influence the epigenetic modification of certain genes (Witt et al., 2018).

## 5 Impact of perinatal vulnerability on oral and craniofacial development

Although they seem to attract relatively less attention, perinatal vulnerabilities have an impact on the development of oral and craniofacial tissues (**Figure 1**).

### 5.1 Development of oral and craniofacial tissues

The developmental process of oral and craniofacial tissues undergoes a sequence of molecular and morphogenetic events, building to form symmetric skeletal structures. Oral and craniofacial development begins with a specific precursor cell type, the neural crest cells (NCCs). A portion of cranial NCCs differentiate into mesenchymal cells *via* epithelial-mesenchymal transition (EMT), and form a framework for oral and craniofacial structures (Sadler, 2018).

The dynamics and activity of NCCs are tightly regulated by a combination of various transcription factors and cytokines, such that the structure does not significantly deviate from species-specific morphology. During development, part of the ectoderm adjacent to the neural plate receives cytokines, including bone morphogenetic proteins (BMPs), fibroblast growth factors (FGFs) and Wnt family molecules. In response to these factors, there is a sequence of transcription factor expression (*e.g.* Msx1/2, Pax3/7, Dlx3/5, Sox9/10, Twist, *etc.*), resulting in the specification of NCC progenitors and their EMT into NCCs (Mishina and Snider, 2014).

Thus, NCCs contribute significantly to major part of skeletal template of oral and craniofacial tissues, and determine the shape of individual faces. The oral and craniofacial bones develop *via* intramembranous or endochondral ossification. The shape and the size of the oral and craniofacial bone are determined by the cytokines (*e.g.* FGFs, BMPs, TGF-β, Wnt proteins and Sonic hedgehog [SHH]) and their downstream signals in skeleton-forming osteoblasts and chondrocytes (Mishina and Snider, 2014; Selleri and Rijli, 2023).

### 5.2 Tooth development

Inside the developing maxilla and mandible, the anlagen of teeth, the tooth germs, begin to grow during gestation. The tooth is a complex organ that consists of tissues of different origins; the enamel is derived from the ectoderm, whereas the dentin and pulp are derived from the ectomesenchyme. Cells of these two origins interact with each other to promote the reciprocal maturation, secreting various types of structural and enzymatic proteins specific or characteristic to the enamel (amelogenin [AMEL], enamelin [ENAM], tuftelin, ameloblastin [AMBN], amelotin [AMTN], odontogenic ameloblast–associated protein [ODAM], MMP-20, kallikrein related peptidase 4 [KLK4] and family with sequence similarity 20, member C [FAM20C]) and dentin (collagens, dentin sialophosphoprotein [DSPP], DSPP derivatives, osteocalcin, osteopontin, dentin matrix protein 1 [DMP1], bone sialoprotein [BSP], matrix extracellular phosphoglycoprotein [MEPE], type II TGF-β receptor interacting protein 1 [TRIP1], glucose regulated protein 78 [GRP78], BMP-1, MMP-2 and MMP-20) (Moradian-Oldak and George, 2021). This developmental process is regulated by cytokines such as Wnt proteins, BMPs, FGFs, SHH and ectodysplasin (Kovacs et al., 2021). The gradation of these factors determines the number and alignment of tooth germs, as well as types and sizes of teeth formed from these germs.

### 5.3 Epigenetic regulation of oral and craniofacial development

Because craniofacial bones constitute the base of individual facial profiles and tooth alignment influences aesthetic perception, regulation of bone formation during gestation determines lifelong facial appearance (Ono and Nakashima, 2022).

#### 5.3.1 Epigenetic regulation of oral and craniofacial bone development

To expand the capacity of the cranium, ossification at the suture should be suppressed from the fetal to childhood stages, but should be promoted during suture fusion. Underlying this stage-switched bone formation is an activation/repression of gene regulation network, which is in part regulated in the epigenetic manner. Various epigenetic factors (summarized in (Shull and Artinger, 2024)) have been reported to be involved in structuring oral and craniofacial bones, and their dysregulation has been reported to trigger diseases by disturbing the expression of transcription factors (*e.g.* Runx2, Sox family molecules and Dlx family molecules) and the subsequent induction of cytokine signaling (*e.g.* BMPs and FGFs) (Shull and Artinger, 2024). A recent study revealed that aberrant activation of a BMP receptor by *Acvr1* gene mutation reduces glycolysis to lower histone lactylation of the *Pdgfra* promoter, leading to failure of the fusion of midline facial processes (Yang et al., 2024).

Even in the absence of these overt genetic mutations, perinatal oxygen and nutrient statuses can profoundly influence craniofacial development through epigenetic reprogramming. Children born with perinatal vulnerabilities exhibit imbalanced facial profiles, with a shorter anterior cranial base and shorter maxillary length as they grow up (Paulsson and Bondemark, 2009), which can be induced by epigenetic dysregulation of skeletogenic cells under such conditions.

#### 5.3.2 Epigenetic regulation of tooth development

During tooth development, there are spatiotemporally specific histone methylation patterns and corresponding expression patterns of histone methyltransferases (Ezh2 and Set7) and demethylases (Kdm5b and Jmjd3) in tooth germs (Zheng et al., 2014) Changes in the epigenetic status of dental pulp cells have been reported to determine their fate toward odontoblasts or osteoblasts (Hu and Fan, 2025). Chromatin architectural proteins, high mobility group N (HMGN)1 and HMGN2 have been reported to suppress the expression of enamel proteins by preventing transcription factors from binding the gene loci of enamel proteins (Januarti et al., 2022). Although these findings indicate that dentinogenesis and amelogenesis can be affected in children with perinatal vulnerability *via* epigenetic regulation, there remains a substantial scope for elucidating the comprehensive mechanisms underlying the epigenetic regulation of tooth development.

### 5.4 Regulation of oral and craniofacial development by mechanical forces

Bone metabolism is profoundly influenced by mechanical forces, such as gravity (Sasaki et al., 2021). Unlike any other skeletal region, the morphology of the oral and maxillofacial bones exhibits striking inter-individual diversity, which is a consequence of genetic, endocrine and mechanical regulation (Ono and Nakashima, 2022).

Fetuses are subjected to mechanical forces during the gestational and perinatal periods in a manner distinct from postnatal life; they are subjected to compressive forces from the uterine wall, but not after birth. Therefore, preterm infants experience less uterine pressure, and instead experience a longer time in incubators in the NICU, which can result in positional cranial deformity (Yang et al., 2019). In addition to cranial bone deformities, maxillary and mandibular bone deformities are more common in preterm birth (Puricelli et al., 2025).

Perinatally vulnerable infants require nutritional and respiratory support through intubation. The tubes are inserted through the mouth and interfere with oral structures, including the palate, tongue and alveolar ridge. Intubation can result in a deep palate, maxillary protrusion, tooth deformity and cross bite (Kim et al., 2019; Oppitz et al., 2021). Intubation can also compromise the development of oral function. Tubes interfere with oral tissues, preventing the acquisition of motor functions such as sucking and swallowing, which provide the mechanical forces necessary for normal jawbone development. Instead, abnormal oral habits are acquired, which may lead to malalignment of teeth and malocclusion. Although direct evidence remains limited, pulmonary or cardiovascular diseases that perturb respiration and cause tachypnea (*e.g.*, patent ductus arteriosus) can lead to low tongue posture and subsequent malalignment of the teeth and malocclusion.

### 5.5 Impact of pharmacological intervention on oral and craniofacial development

In addition to mechanical interventions, infants in the NICU often require pharmacotherapy that can affect their development. Although the routine use of systemic postnatal steroids (PNS) is not recommended due to safety concerns, they remain a necessary intervention for the prevention or treatment of bronchopulmonary dysplasia (BPD), by ameliorating inflammation and fibrosis (Rohr et al., 2014). Similarly, loop diuretics are frequently used in the NICU to improve pulmonary function (O'Brien et al., 2024). However, these drugs are reported as risk factors for metabolic bone diseases, restricting cranial bone growth (Alsaif et al., 2025). Therefore, given the systemic suppression of bone formation, it is theoretically suggested that these drugs also compromise the development of oral and craniofacial bones and teeth.

## 6 Treatment of children with perinatal vulnerabilities

Infants with perinatal vulnerabilities are typically shorter and lighter at birth than their full-term or normal-birth-weight peers. However, these infants often grow rapidly to catch up with population norms. Although relatively uncommon, oral and craniofacial tissues can be affected by the vulnerabilities and can catch up as well. *Note: the term “catch-up growth” in this chapter encompasses both acceleration of physiological growth and correction of deformity.*

### 6.1 Catch-up growth of oral and craniofacial structures

Unlike other organs, including the brain, heart, lung and kidney, the oral and craniofacial complex is predominantly composed of hard tissues (bone and teeth). Because these hard tissues determine head and facial morphology, aesthetic outcomes must be explicitly considered. The architecture of the bone cannot be corrected pharmacologically at present; thus, catch-up growth in this region critically depends on controlled external mechanical forces, with surgical intervention when necessary. Teeth do not undergo metabolic processes as observed in the bone. Therefore, catch-up growth does not occur after the completion of tooth formation.

### 6.2 Cranial orthosis for positional cranial deformity

Infants with perinatal vulnerabilities are at increased risk of positional cranial deformities because their underdeveloped neck muscles prevent them from rolling. Consequently, the cranial bone surface in contact with the bed remains in prolonged contact, leading to unilateral flattening of the cranium (Chrenko et al., 2025). Because the suture and fontanelle of the cranium do not close and bone fragments are mobile, this asymmetric morphology is usually alleviated as infants grow up to acquire the motor function of the rollover.

In cases where there is moderate or severe asymmetry, a cranial orthosis is applied between 4 and 7 months of age, so that the cranium becomes symmetric and neurological problems are avoided. The efficacy of cranial orthosis has been assessed in several studies. Most concluded that this therapy was efficient in catching up with symmetrical cranial shapes. However, some studies did not find any significant improvements. This discrepancy may be attributed to differences in the ethnic background and age of the infants (Takamatsu et al., 2021; van Wijk et al., 2014).

The fusion of cranial sutures can occur as early as the gestational period or during infancy, even in the absence of identifiable genetic mutations (*i.e.*, non-syndromic craniosynostosis). Although the underlying mechanisms remain unclear, perinatal vulnerabilities have been associated with an increased risk of craniosynostosis (Sanchez-Lara et al., 2010). Surgical intervention is performed within one year of age to mitigate the risk of elevated intracranial pressure and to secure adequate space for brain growth (Faasse et al., 2023).

### 6.3 Orthodontic treatments for correcting tooth alignment

**Figure 2:**
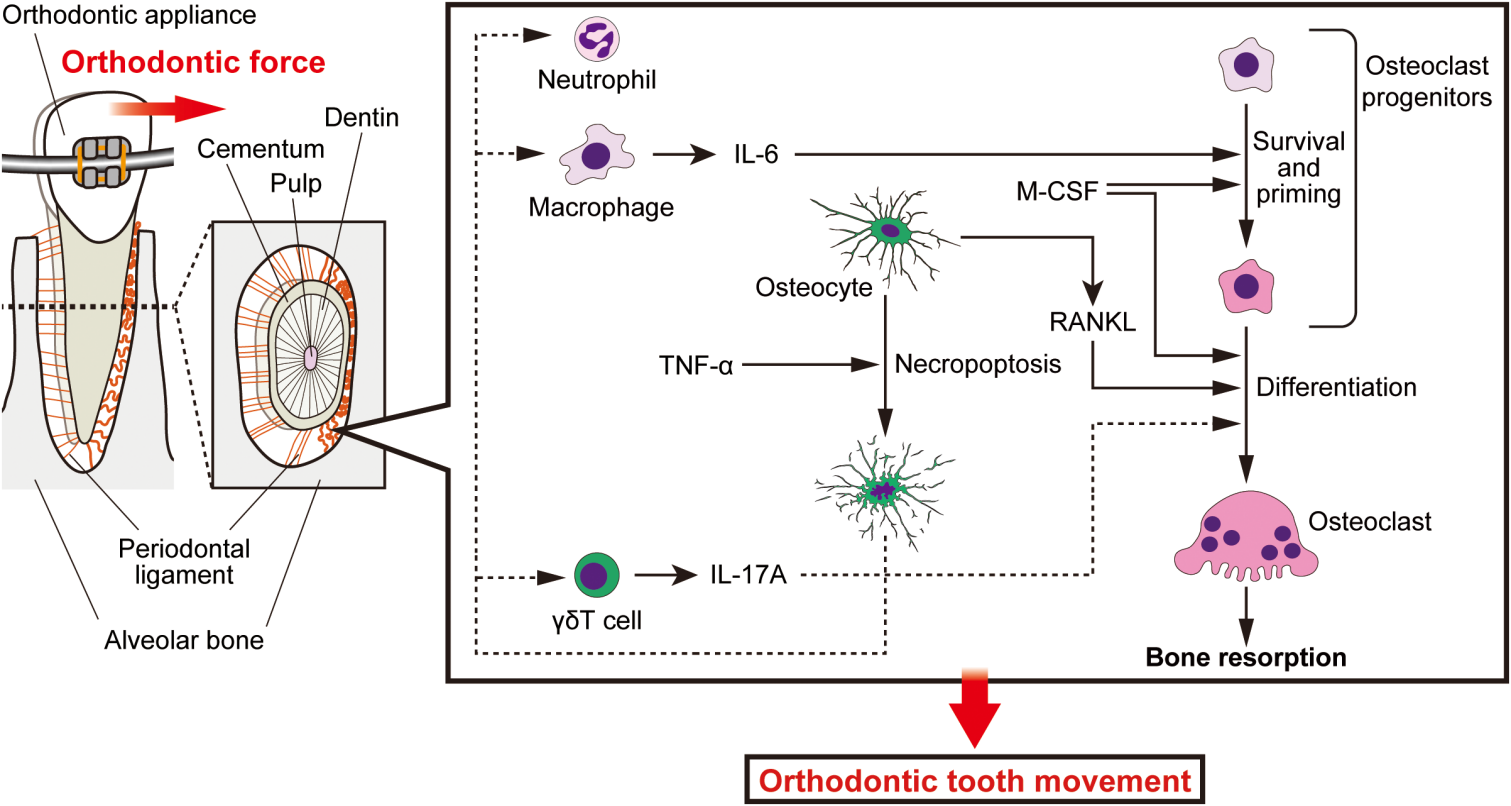
Cellular and molecular biology of orthodontic tooth movement. Orthodontic tooth movement is achieved through the remodeling of the alveolar bone. Orthodontic intervention increases innate immune cells in the periodontal ligament space. Macrophages produce IL-6, which promotes the survival of osteoclast progenitor cells and engages them toward the osteoclast lineage, together with M-CSF. These progenitors undergo osteoclastogenesis upon stimulation with M-CSF and RANKL produced by osteocytes. TNF-α induces the necroptosis of osteocytes. It is suggested that the osteocyte death stimulates osteoclastogenesis by driving innate immunity. γδT cells in the periodontal tissue produce IL-17A and enhance tooth movement. Collectively, these cellular and molecular events integrate mechanical forces, inflammation and cell death to facilitate orthodontic tooth movement.

Prolonged NICU hospitalization can compromise the structure and function of oral and maxillary tissues in infants. Tooth misalignment can be corrected using an orthodontic approach. Orthodontic treatment utilizes bone remodeling induced by the mechanical loading on the alveolar bone surrounding the tooth roots. The compression side of the alveolar bone undergoes bone resorption, whereas bone formation occurs on the tension side, the underlying mechanisms of which are summarized in **Figure 2**.

As can be presumed by the fact that orthodontic tooth movement (OTM) coincides with periodontal pain, orthodontic intervention induces inflammation in the periodontal tissues: macrophages, γδT cells and their related cytokines in the periodontal tissues (Wald et al., 2021; Xu et al., 2022). Pro-inflammatory cytokines are known to stimulate mesenchymal cells to express the key osteoclastogenic cytokine, receptor activator of NF-κB ligand (RANKL). Interleukin (IL)-6 primes macrophages there to be sensitive to macrophage colony-stimulating factor (M-CSF) and RANKL (Toyama et al., 2023). IL-17A can induce both bone resorption and formation (Komatsu et al., 2014; Ono et al., 2016); thus, it is possible for this cytokine to induce bone resorption at the compression side and formation at the tension side. Osteoclast progenitors on the alveolar bone surface receive RANKL from osteocytes, which can sense and respond to mechanical forces, and differentiate into osteoclasts (Shoji-Matsunaga et al., 2017). TNF-α leads to osteocyte necroptosis at the compression side, which may stimulate osteoclastogenesis by stimulating innate immune cells with apoptotic cell components (Ohori et al., 2025).

Children with perinatal vulnerabilities often experience difficulty in gaining the ability to feed orally; instead, they may acquire parafunctional habits, which can lead to a relapse of tooth alignment after orthodontic treatment. To prevent relapses, as well as to acquire normal oral functions, many types of myofunctional therapies (MFTs) are conducted in clinics (Chang et al., 2022; Mahmoodabadi et al., 2024).

### 6.4 Orthognathic treatments for correcting the oral and maxillary tissues

**Figure 3:**
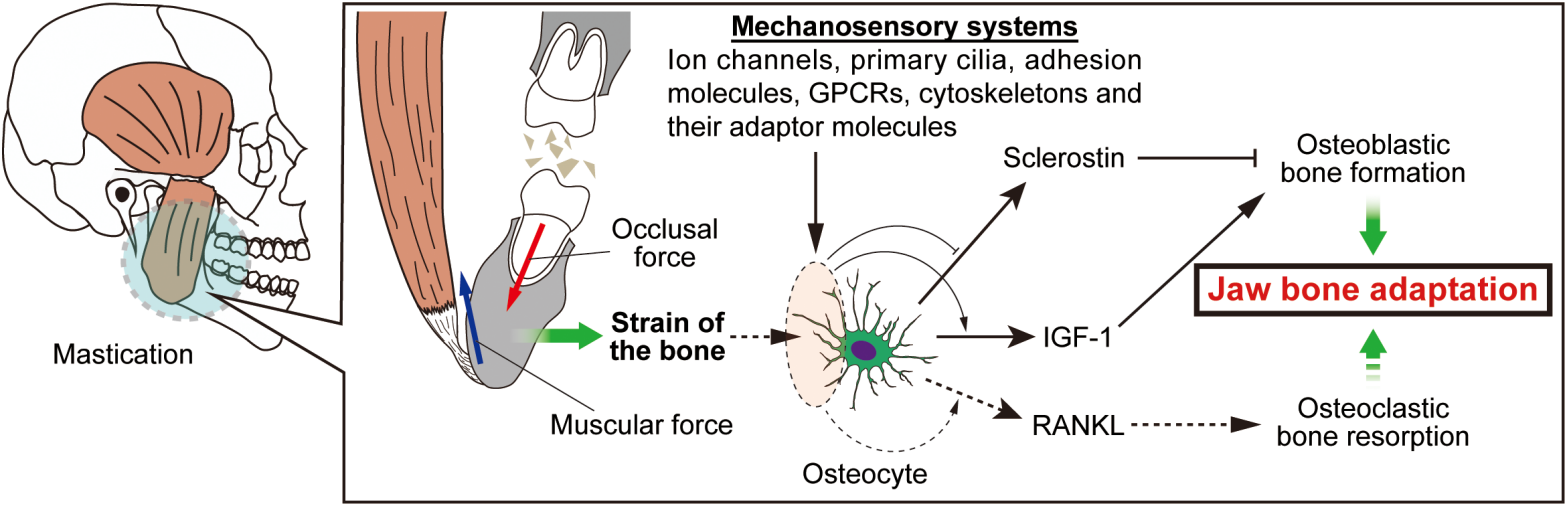
Cellular and molecular biology of jawbone adaptation to mechanical loading. During mastication, muscular and occlusal forces are loaded onto the jawbone, thereby generating strain on the bone architecture. The strain is sensed by mechanosensory molecules of bone-constituting cells. Osteocytes function as the major mechanosensory cells in the bone tissue. Forceful mastication suppresses sclerostin production and increases IGF-1 production, together promoting osteoblastic bone formation at the masseteric ridge. Osteocytes may produce RANKL during mastication, inducing osteoclastic bone resorption as part of the resorptive bone formation process. This bone metabolic process alters the shape of the jawbone such that it can withstand masticatory forces.

Although the shape of the mandible is predominantly governed by genetic factors, the mechanical loading on the bone has a significant influence on its shape and quality. Animal models have shown that reduced mastication results in a decrease in bone mineral density of the mandible (Fukushima-Nakayama et al., 2017; Hichijo et al., 2015). On the other hand, forceful mastication during the growth period stimulates appositional bone formation at the attachment of the masseter muscle to withstand the masticatory force, the underlying mechanisms of which are summarized in **Figure 3**.

In response to forceful masticatory force, osteocytes in the mandibular angle produce more insulin-like growth factor (IGF)-1 and less sclerostin, resulting in osteoblastic differentiation of mesenchymal cells in the tendon of the masseter muscle and subsequent bone formation at the site (Inoue et al., 2019). As osteocytes in the jawbone express RANKL, it has been speculated that osteocyte RANKL may be involved in bone metabolism upon masticatory loading onto the jawbone, creating a concave structure (resorptive bone modeling) (Seeman and Martin, 2019). Mechanical loading on the bone is sensed by a variety of machineries including ion channels. In particular, osteocyte Piezo1 and Piezo2 are recognized as the major channels involved in bone metabolism. However, these channels are likely to participate in as early development as embryonic stage of the jawbone, and are not significantly involved in the later developmental stages, in which mechanical loading has a greater influence on bone metabolism than in early stages (Nie et al., 2024; Nottmeier et al., 2023). Thus, there is a large room for investigation into the underlying mechanisms. Bone-constituting cells, especially osteocytes, harbor many other types of mechanosensory systems that can regulate jawbone metabolism under mechanical loading (Wang et al., 2022). Collectively, masticatory loading may offer a window for enhancing mandibular robustness in preterm infants with compromised bone quality. As mechanical loading on the locomotorium (bone and muscle) is mimicked pharmaceutically (Ono et al., 2022), future drug therapies may enable induced catch-up growth of the mandible.

**Figure 4:**
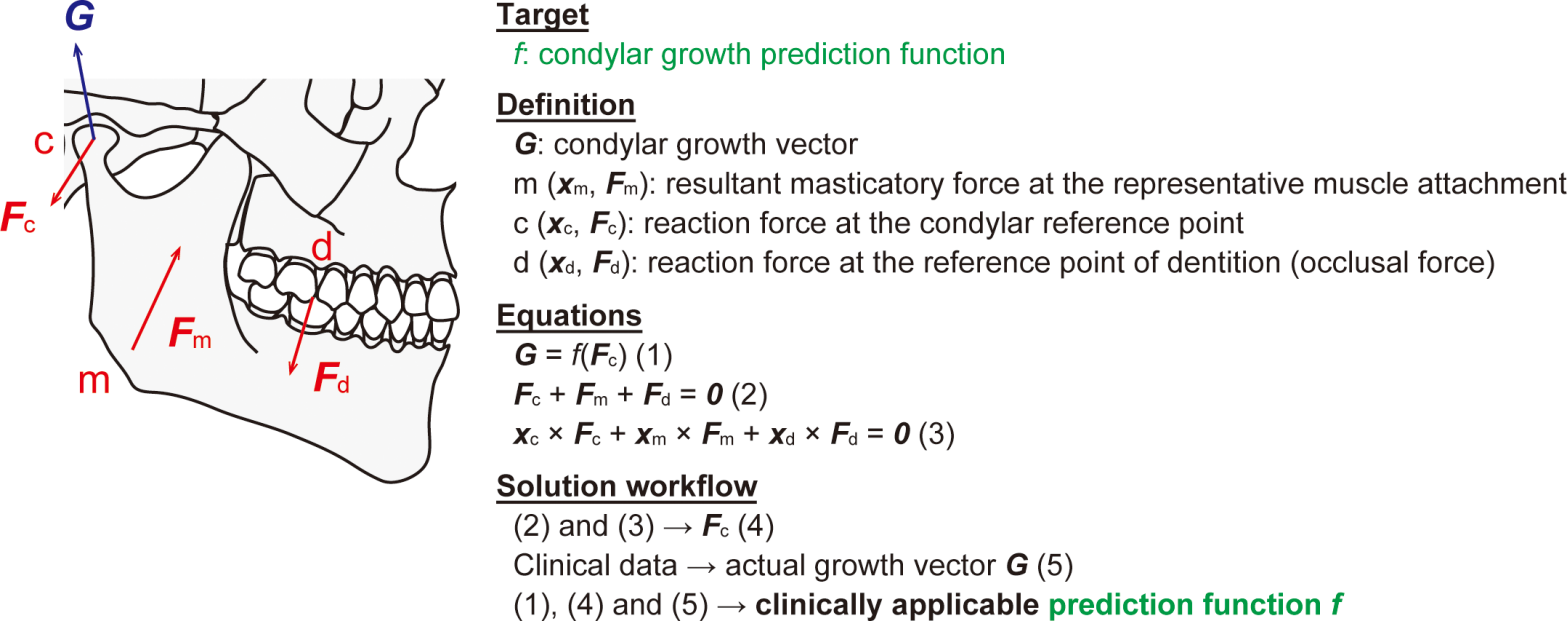
Simulation of the mandibular growth at the condyle (*hypothesis by the author*). Mandibular growth plays a pivotal role in determining the shape and function of the oral and maxillofacial complex. Therefore, it has long been regarded as a central challenge to determine the condylar growth prediction function, which is expressed as *f* in this figure. Mandibular growth at the condyle is known to be influenced by the mechanical force generated in the condyle (***F***_c_) in response to mastication and occlusion. Thus, the growth vector (***G***) can be expressed as ***G*** = *f*(***F***_c_) (1). When treated as a rigid body, the mandible exhibits no net translation or rotation during occlusion, in which the bone is subjected to the resultant masticatory force ***F***_m_ at ***x***_m_ and its reaction forces at the dentition (***F***_d_ at ***x***_d_, also called occlusal force) and at the condyle (***F***_c_ at ***x***_c_). This is expressed as ***F***_c_ + ***F***_m_ + ***F***_d_ = ***0*** (2) and ***x***_c_ × ***F***_c_ + ***x***_m_ × ***F***_m_ + ***x***_d_ × ***F***_d_ = ***0*** (3). Simultaneously solving Equations (2) and (3) yields ***F***_c_ (4). The actual growth vector ***G*** is measured by superimposing serial computed tomography (CT) scans in clinical cases (5). By combining Equations (1), (4) and (5), a clinically applicable growth prediction function, *f*, can be derived. ***x***, position vector; ***F***, force vector; ***0***, zero vector; ***x*** × ***F***, cross product of the vectors.

The mandibular condyle is a component of the temporomandibular joint and the predominant site of endochondral growth of the ramus. The direction and magnitude of mandibular condylar growth have long been recognized to be profoundly influenced by functional masticatory stimuli (Enomoto et al., 2010; Sella-Tunis et al., 2018). Although these findings theoretically suggest the potential of targeted masticatory loading as an approach to treat micrognathia and related jaw deformities, such strategies have not been translated into clinical practice; instead, orthodontic and orthognathic procedures, including osteotomy, are usually adopted (Borzabadi-Farahani, 2025). The primary obstacle to adopting masticatory loading as a therapeutic modality for jaw deformities may lie in the fact that, despite pioneering and valuable attempts (Koseki et al., 2008), no fully reliable method has yet been established to quantitatively predict the direction and magnitude of condylar growth induced by masticatory or occlusal forces in humans. Therefore, ongoing advances in mechanobiology, real-time growth monitoring and personalized biomechanical simulations are expected to overcome these limitations (**Figure 4**).

### 6.5 Restoration of teeth exhibiting tooth malformation

Tooth formation in children with perinatal vulnerabilities is frequently compromised simultaneously in multiple teeth, manifesting as amelogenesis imperfecta, dentinogenesis imperfecta, dentin dysplasia or molar-incisor hypomineralization (MIH). Affected teeth exhibit discoloration, compromised surface integrity, accelerated attrition/abrasion and increased susceptibility to caries and fractures due to morphological anomalies and defective mineralization (Barron et al., 2008; Crawford et al., 2007; de La Dure-Molla et al., 2015; Goldberg, 2019). In severe cases, persistent hypersensitivity and chronic pain may profoundly impair mastication, speech and psychosocial development, underscoring the urgency for comprehensive interventions, including high-concentration fluoride applications, resin infiltration, direct restorations and prostheses tailored to age and severity (Goud and Deshpande, 2011; Hoogeveen et al., 2025; Wang et al., 2024a). Compared to the general population, these conditions simultaneously affect numerous teeth with a significantly shortened tooth lifespan, often necessitating early prosthetic intervention (Barron et al., 2008; Crawford et al., 2007). In the long term, regenerative approaches involving gene therapy are anticipated to offer the most promising curative strategy. Because masticatory function has been linked to cognitive performance and brain development, early restoration of adequate occlusion and masticatory efficiency may be critical not only for oral health but also for optimizing neurocognitive outcomes in this vulnerable population (Alvarenga et al., 2019; de Siqueira Mendes et al., 2021; Fukushima-Nakayama et al., 2017).

## 7 Discussion

Children born with perinatal vulnerabilities represent a rapidly growing population due to advances in neonatal care. Sustained clinical observations and advances in basic research have increasingly elucidated both the phenotypic spectrum and molecular mechanisms.

Recently, assisted reproductive technology (ART) has brought hope to countless couples struggling with infertility. However, it has also contributed to a higher incidence of PTB and LBW, although perinatal outcomes continue to improve (Wennerholm and Bergh, 2020). As the global cohort of ART-conceived individuals reaches adulthood in the coming decades, clinical insights and management strategies developed for children with perinatal vulnerabilities will become applicable to this population.

Large for gestational age (LGA) is another facet of perinatal complications, situated at the opposite end of the fetal growth spectrum from SGA and LBW (**Table 1**). Although there is a higher risk of metabolic diseases in LGA (Hong and Lee, 2021; Johnsson et al., 2015), the volume of research on the long-term consequences of LGA remains considerably smaller than that on perinatal vulnerabilities. When managing patients with a history of LGA who develop these metabolic conditions, extensive clinical experience and evidence from patients born with perinatal vulnerabilities are likely to provide useful guidance.

Notably, oral and craniofacial tissues are sensitive to perinatal vulnerabilities, manifesting as deformities of the cranial bones, jawbones and teeth. In stark contrast to NCDs, which predominantly affect metabolic systems, hard tissue deformities are not reversible, and dentists and surgeons must currently rely on invasive procedures such as plastic and orthognathic surgeries, orthodontic treatment, tooth restoration and prosthetic treatments to correct the morphology and function of the hard tissues. Importantly, however, these anomalies are often tangible and can be recognized earlier than extracranial sequelae; thus, they may serve as accessible indicators for predicting extracranial sequelae that remain latent.

Because bone development is regulated by mechanical forces, intervention therapies for mastication and occlusion represent a theoretically promising complementary approach for correcting jawbone deformities. Such biologically guided orthognathic strategies have not yet been sufficiently developed for routine clinical applications, and rapid advances in mechanobiology and personalized simulation hold considerable potential for future translation.

Teeth are unique tissues that cannot regenerate themselves and, therefore, must be treated with artificial materials, including resins and metals. Because these materials cannot fully emulate natural teeth, patients with tooth anomalies resulting from perinatal vulnerabilities require repeated replacement or modification of restorations throughout their growth (*e.g.*, dental implants should not be applied before the cessation of the growth period; instead, temporary restorations are applied). Regenerative approaches involving gene therapy to generate fully functional teeth in these patients would be the ultimate solution and are strongly desired.

## 8 Conclusions

Perinatal vulnerabilities profoundly compromise the development of oral and craniofacial hard tissues, as well as extracranial organs. However, the mode of disorders, catch-up growth patterns and their therapies are completely distinct from extracranial complications, such as metabolic diseases: the resulting hard-tissue defects and deformities are irreversible, and truly therapeutic treatments remain under development. The prevalence of survivors of perinatal vulnerabilities (including those who have been born with ART) keeps increasing. Therefore, it is imperative that robust collaborative networks spanning all healthcare providers be established to provide the patients with seamless, sequential and comprehensive care, while simultaneously raising awareness among healthcare providers, patients and their families.
